# Gene Targeting of the Cysteine Peptidase Cathepsin H Impairs Lung Surfactant in Mice

**DOI:** 10.1371/journal.pone.0026247

**Published:** 2011-10-12

**Authors:** Frank Bühling, Martin Kouadio, Caroline E. Chwieralski, Ursula Kern, Jens M. Hohlfeld, Nicole Klemm, Nicole Friedrichs, Wera Roth, Jan M. Deussing, Christoph Peters, Thomas Reinheckel

**Affiliations:** 1 Institute of Laboratory Medicine, Carl-Thiem-Klinikum, Cottbus, Germany; 2 Institut of Molecular Medicine and Cell Research, Albert-Ludwigs-University, Freiburg, Germany; 3 Insitute of Molecular and Clinical Immunology, Otto-von-Guericke University, Magdeburg, Germany; 4 Faculty of Biology, Albert-Ludwigs-University, Freiburg, Germany; 5 Spemann Graduate School of Biology and Medicine (SGBM), Albert-Ludwigs-University, Freiburg, Germany; 6 Division of Immunology, Allergology and Airway Research, Fraunhofer Institute of Toxicology and Experimental Medicine, Hannover, Germany; 7 BIOSS Centre of Biological Signalling Studies, Albert-Ludwigs-University, Freiburg, Germany; University of South Florida College of Medicine, United States of America

## Abstract

**Background:**

The 11 human cysteine cathepsins are proteases mainly located in the endolysosomal compartment of all cells and within the exocytosis pathways of some secretory cell types. Cathepsin H (Ctsh) has amino- and endopeptidase activities. *In vitro* studies have demonstrated Ctsh involvement in the processing and secretion of the pulmonary surfactant protein B (SP-B). Furthermore, Ctsh is highly expressed in the secretory organelles of alveolar type II pneumocytes where the surfactant proteins are processed.

**Methodology/Principal Findings:**

Hence, we generated Ctsh null mice by gene targeting in embryonic stem cells to investigate the role of this protease in surfactant processing *in vivo*. The targeting construct contains a ß-galactosidase (lacZ) reporter enabling the visualisation of Ctsh expression sites. Ctsh-deficiency was verified by northern blot, western blot, and measurement of the Ctsh aminopeptidase activity. *Ctsh* ^−/−^ mice show no gross phenotype and their development is normal without growth retardation. Broncho-alveolar lavage (BAL) from *Ctsh* ^−/−^ mice contained lower levels of SP-B indicating reduced SP-B secretion. The BAL phospholipid concentration was not different in *Ctsh^+/+^* and *Ctsh* ^−/−^ mice, but measurement of surface tension by pulsating bubble surfactometry revealed an impairment of the tension reducing function of lung surfactant of *Ctsh* ^−/−^ mice.

**Conclusions/Significance:**

We conclude that cathepsin H is involved in the SP-B production and reduced SP-B levels impair the physical properties of the lung surfactant. However, Ctsh defiency does not reproduce the severe phenotype of SP-B deficient mice. Hence, other proteases of the secretory pathway of type II pneumocytes, i.e. cathepsins C or E, are still able to produce surfactant of sufficient quality in absence of Ctsh.

## Introduction

The family of papain-like cysteine proteases (Clan CA, C1 family) consists of 11 members in humans, i.e. cathepsins B, C (J, dipeptidyl peptidase I), F, H, K (O, O2), L, O, S, V (L2), W (lymphopain), and X (P, Y, Z) [1]. All of these proteases have orthologous enzymes in mice, only murine cathepsin L is homologous to both human cathepsin L and human cathepsin L2(V) [2], [3]. Hence the analysis of mouse models with targeted inactivation of cysteine cathepsins has been widely used as rational approach to elucidate the *in vivo* functions of these endosomal/lysosomal enzymes that can also function outside the cell and as truncated protease variants in the cytosol and the nucleus [4], [5]. Specific cysteine cathepsins are involved in precursor protein activation (including proenzymes and prohormones), MHC-II-mediated antigen presentation, bone remodelling, keratinocyte differentiation, hair cycle, reproduction, and apoptosis [6]. They have also been implied to participate in tumor progression and metastasis as well as in inflammatory diseases, such as inflammatory rheumatoid arthritis, atherosclerosis, and periodontitis, and are potential therapeutic targets [7].

Mature Ctsh is a 25 kDa endosomal/lysosomal enzyme that has been activated by proteolytic removal of a 75 amino acid propeptide from the Ctsh-zymogene. Cathepsin H and Cathepsin B are unique among lysosomal cysteine proteases in that they are both an exopeptidase and an endopeptidase [8], [9], [10]. The structural basis of the aminopeptidase activity of Ctsh is provided by an eight residue residual portion of the propeptide, termed mini-chain, that remains attached to the papain-like structure by a disulfide link and provides its C-terminal Thr 83P carboxylic group to capture the positively charged N-terminal amino group of substrate proteins [11]. Removal of the mini-chain renders Ctsh a complete endoproteinase [12], [13]. However, it has been experimentally shown that Ctsh is able to execute specific endoproteolytic cleavage in native substrate proteins even in the presence of the mini-chain [14].

Cathepsin H is considered a ubiquitously expressed protease; however, strong expression has been reported in type II pneumocytes [15]. Interestingly cathepsin C, a strict aminopeptidase, and Ctsh are the only cysteine-cathepsins found in the secretory compartment of type II pneumocytes, and both proteases have been shown to be functionally redundant [15], [16]. The lamellar and multivesicular bodies of these cells produce the lung surfactant that is essential to reduce surface tension at the air-liquid interface within the alveoli thus enabling breathing mechanics and gas exchange. Pulmonary surfactant is composed of phospholipids and proteins. These surfactant proteins (SP) are either hydrophilic glycoproteins, i.e. SP-A and SP-D, or very hydrophobic, i.e. SP-B and SP-C [17]. Notably, hereditary SP-B deficiency results in neonatal onset surfactant deficiency and respiratory failure in humans and mice [18]. The ∼8.7 kDa SP-B found in the air-space is generated from ProSP-B by several steps of limited proteolysis within the secretory compartment of type II pneumocytes. SP-B processing in type II pneumocytes is sensitive to treatment with cysteine-cathepsin inhibitors and *in vitro* studies proved that Ctsh is capable of processing SP-B [14], [19]. Since a mouse model for Ctsh-deficiency has not been available, we generated a Ctsh knock-out mouse model in order to address the role of Ctsh in SP-B processing and surfactant generation *in vivo*.

## Results and Discussion

**Figure 1:**
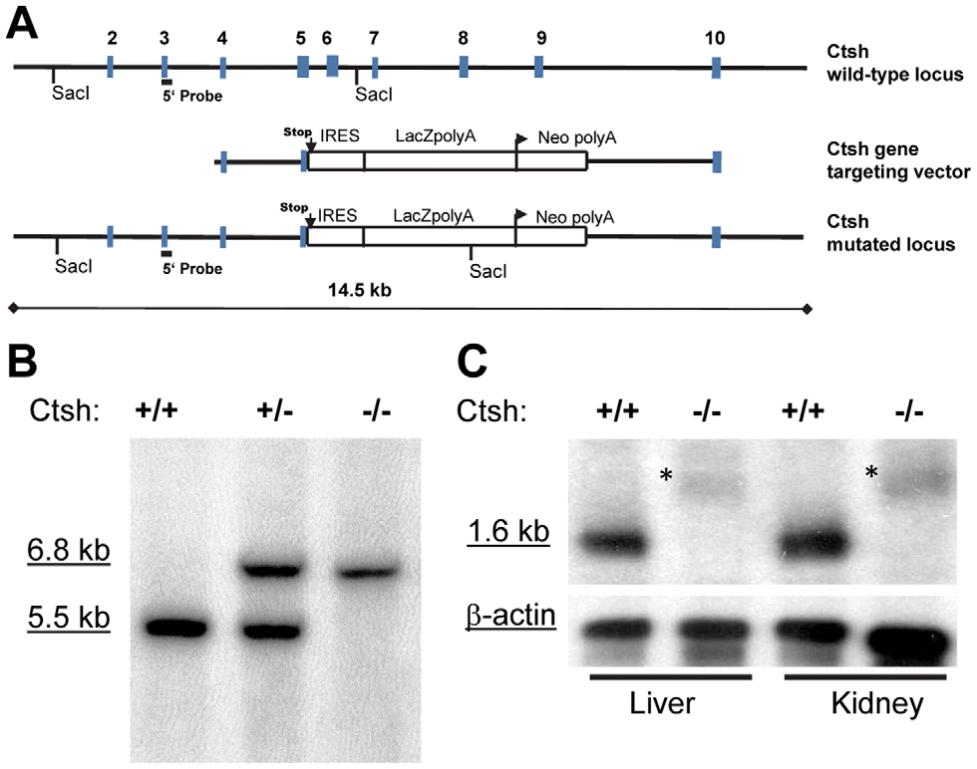
Targeted disruption of the cathepsin H (Ctsh) gene. (**A**) Scheme for the targeted disruption of mouse Ctsh gene by homologous recombination. Exons are indicated by number. (**B**) Southern blot analysis of SacI-digested genomic DNA from mouse liver by the 5′ external probe denoted in panel A. Expected fragment sizes are 5.5 kb for wild-type and 6.8 kb for mutant *Ctsh* alleles. (**C**) Northern blots from liver and kidney samples of *Ctsh^+/+^* and *Ctsh* ^−/−^ mice. The Ctsh 5′ probe detects the genuine 1.6 kb mouse Ctsh transcript in the *Ctsh^+/+^* samples. *Denotes an enlarged transcript in *Ctsh* ^−/−^ consisting of Ctsh exons 1–5 plus lacZ reporter.

The murine cathepsin H (Ctsh) gene is located on mouse chromosome 9 comprising 12 exons that span a genomic region of 22 kb. In the Ctsh gene targeting vector a β-geo cassette containing IRES, lacZ-Reporter and a neomycine (neo) resistance was flanked at the 5′ end by approximately 2 kb Ctsh sequence containing exon 4 and part of exon 5 and a DNA fragment at the 3′ end consisting of intron 9 and exon 10 (Fig. 1A). Hence, by homologous recombination in HM-1 mouse embryonic stem cells part of exon 5 through part of intron 9 (including the active site cysteine critical for enzyme activity in exon 6) of the Ctsh gene was deleted and substituted by the β-geo cassette (Fig. 1A). Southern blot using SacI restriction digest and probing with the external 5̀probe in genomic tail DNA confirmed correct homologous recombination in the *Ctsh* locus by showing the expected 1.3 kb band shift (Fig. 1B). These results were further confirmed by long range PCR with external and internal primers (data not shown). Northern blots with the 5′ probe binding to exon 3 revealed absence of the 1.6kb mRNA of Ctsh in liver and kidney of *Ctsh* ^−/−^ mice (Fig. 1C). In the *Ctsh* ^−/−^ samples a larger band corresponding to the fusion transcript comprising the first 5 exons of Ctsh and the IRES-*LacZ* reporter was detected (Fig. 1C). Taken together, these results proof correct homologous recombination of the *Ctsh* locus and the functionality of the targeting construct.

**Figure 2:**
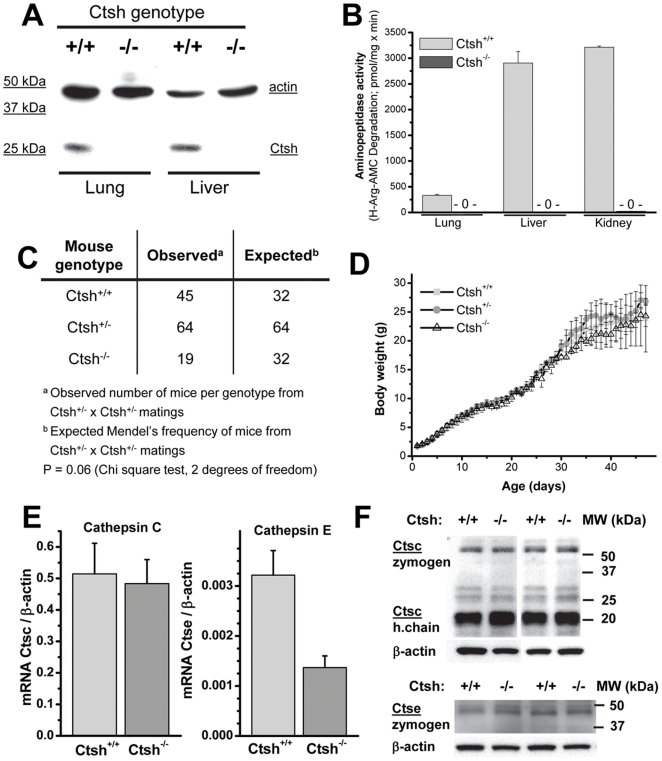
Cathepsin expression and gross phenotype of Ctsh-deficient mice. (**A**) Western blots for Ctsh detection in lungs and liver of *Ctsh^+/+^* and *Ctsh* ^−/−^ mice. (**B**) Detection of “acidic” aminopeptidase activity at pH 6.0 in lungs, livers and kidneys of f *Ctsh^+/+^* and *Ctsh* ^−/−^ mice (n = 3). (**C**) Observed and expected genotype frequencies of litters from *Ctsh* ^+/−^ x *Ctsh* ^+/−^ matings. (**D**) Weight gain of female littermates from heterozygous matings (n = 5 per genotype). (**E**) mRNA expression of cathepsin C (Ctsc) and cathepsin E (Ctse) measured by quantitative ‘real-time’ RT-PCR in lungs of *Ctsh^+/+^* and *Ctsh* ^−/−^ mice (n = 5 per group). (**F**) Cathepsin C (Ctsc) and cathepsin E (Ctse) detected by Western blotting in *Ctsh^+/+^* and *Ctsh* ^−/−^ lung lysates.

Accordingly, tissues of *Ctsh* ^−/−^ mice do not express the protease as a protein (Fig. 2A) and show abolished proteolysis of the *Ctsh*-specific aminopeptidase substrate H-Arg-AMC (Fig. 2B). Breeding of heterozygous mice produced offspring with a statistical trend (p = 0.06) towards a reduced number of Ctsh null mice as compared to the expected Mendelian ratio (Fig. 2C). However, Ctsh deficient mice do not show a gross morphological phenotype. Furthermore, Ctsh null mice show no impairment of reproductive capacity or breeding behavior or nursing, and can be maintained as homozygous mutant mouse line. Ctsh^−/−^ and Ctsh^+/−^ mice developed indistinguishably from their wild-type littermates, reached normal body weight (Fig. 2D), and lived without phenotype for observation periods of up to two years. Histomorphology of kidney, liver, heart, brain, thymus, and spleen of *Ctsh* ^−/−^ mice did not show any signs of pathology (data not shown). Cathepsin C (Ctsc; also named dipeptidylpeptidase 1) is an aminopeptidase that has been shown to cooperate with Ctsh in the processing of pro-granzyme B [16]. The cathepsin D-like aspartic protease cathepsin E (Ctse) is another protease that might compensate for the loss of Ctsh in the knock-out [20]. Hence we determined the expression of both proteases as mRNA and protein (Fig. 2E, F). Ctsc was not differently expressed or processed in lung tissue samples from *Ctsh^+/+^* and *Ctsh* ^−/−^ mice. In general Ctse expression in the lungs was at the limit of detection. Nevertheless, we found an about 50% reduction of Ctse mRNA levels in *Ctsh* ^−/−^ (Fig. 2E). However, Western Blots for Ctse could not verify a major difference in Ctse protein levels between *Ctsh^+/+^* and *Ctsh* ^−/−^ lungs (Fig. 2F). Hence, our data exclude a compensatory upregulation of cathepsins C or E in the lungs of Ctsh deficient mice. However, both proteases may be able to functionally compensate for the absence of Ctsh simply by being able to sufficiently cleave most of the substrate repertoire of Ctsh.

**Figure 3:**
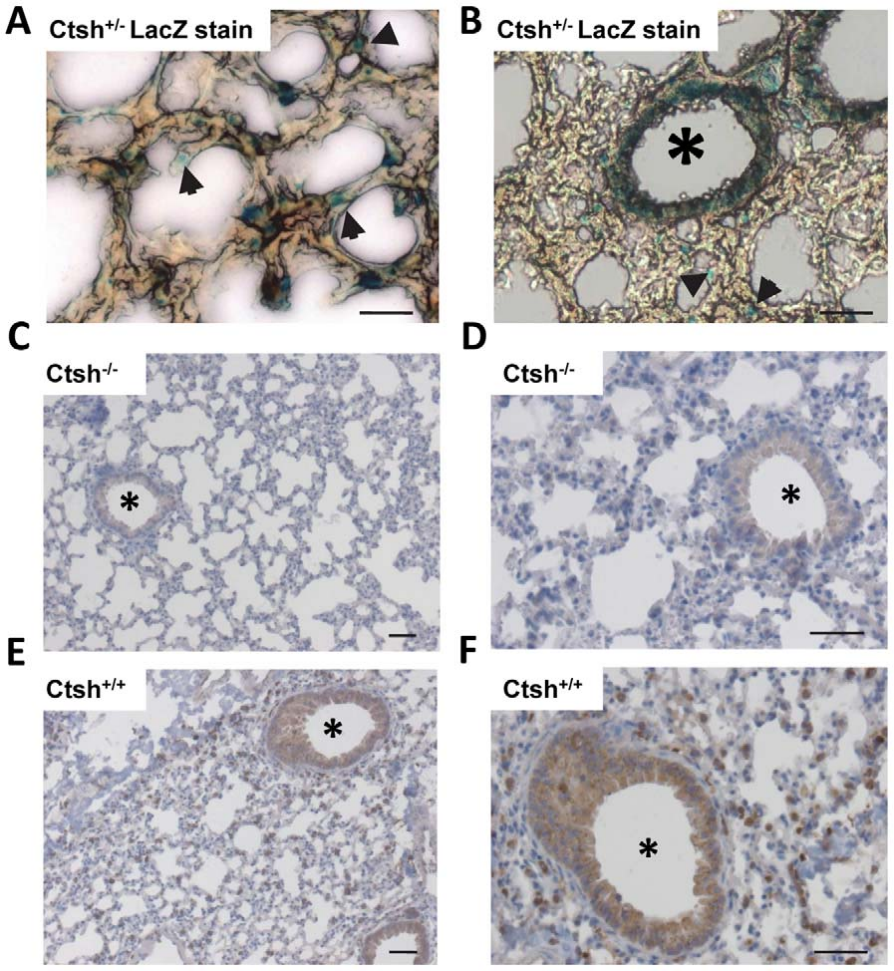
Cathepsin H expression in the lung. (**A**) Expression of the lacZ reporter (blue staining) in *Ctsh* ^+/−^ mice as indication for Ctsh transcription in distinct alveolar cells, i.e. type II pneumocytes (arrows heads), and (**B**) in small airway bronchioles (indicated by *). (**C**–**D**) Histology and absent immunohistochemical detection of Ctsh in *Ctsh* ^−/−^ lungs. (**E–F**) Immunohistochemical detection of Ctsh in lungs of wild-type mice confirms the expression of Ctsh in distinct pneumocytes and bronchiolar epithelium (indicated by *). Scale bars are 50 µm.

In the lung Ctsh has been shown to be specifically expressed in type II pneumocytes [15]. Taking advantage of the *lacZ* reporter in the Ctsh targeting construct a selective expression was detected in distinct alveolar cells in lungs of *Ctsh* ^+/−^ mice with the typical appearance of type II pneumocytes (Fig. 3A). In addition, considerable lacZ expression was detected in the epithelia of small airway bronchioles (Fig. 3B). Involvement of Ctsh in lung branching morphogenesis has been recently reported [21], however, lung morphology and histology was not altered in Ctsh-deficient mice (Fig. 3C, D). Immunohistochemical detection of Ctsh confirmed the absence of the protease in the lungs of Ctsh^−/−^ mice (Fig. 3C,D), while in wild-type mice the Ctsh staining pattern in lung tissue was confined to specific alveolar cells, i.e. type II pneumocytes, and to bronchioles (Fig. 3E,F). This provides evidence for the correct function of the LacZ reporter that has been introduced to the Ctsh locus by gene targeting (compare Fig. 1A).

**Figure 4:**
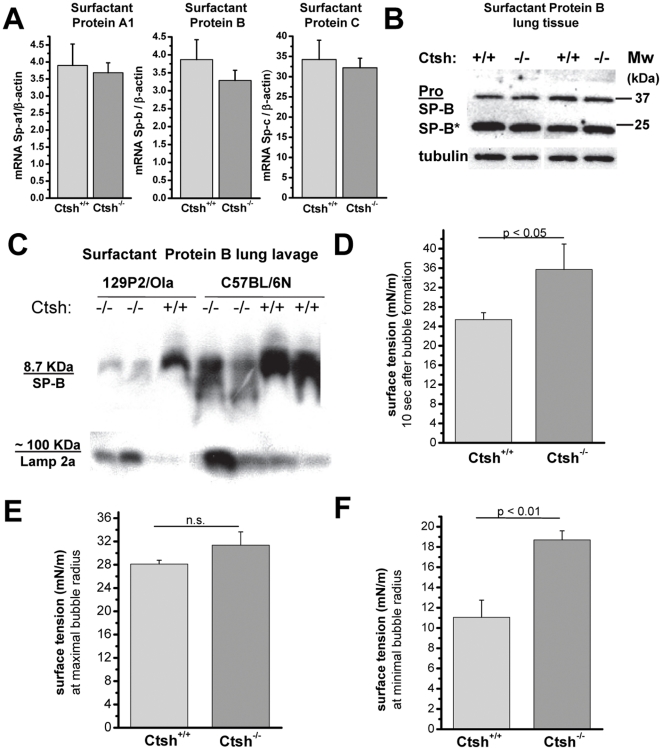
Cathepsin H function in production of lung surfactant proteins. (**A**) mRNA expression of surfactant proteins A1, B, and C measured by quantitative ‘real-time’ RT-PCR in lungs of *Ctsh^+/+^* and *Ctsh* ^−/−^ mice (n = 5 per group). (**B**) Detection of surfactant protein B (SP-B) in lung tissue lysates (**C**) Western blot detection of SP-B in broncho-alveolar lavage (BAL) of *Ctsh^+/+^* and *Ctsh* ^−/−^ mice of 2 genetic backgrounds (129P2/OlaHsd and C57BL/6N). The lysosomal membrane associated protein 2a (Lamp 2a) is present at the limiting membrane of lamellar bodies [27], [28] and serves as loading control independent of the surfactant proteins. (**D**–**F**) Surface activity of BAL fluid measured by pulsating bubble surfactometry (n = 6–10).

Since type II pneumocytes produce and secrete surfactant we analyzed the mRNA expression of the surfactant proteins A1 (SP-A1), B (SP-B), and C (SP-C) (Fig. 4A). The mRNA levels of all three surfactant proteins were not altered in the lungs of *Ctsh* ^−/−^ mice. Previous studies *in vitro* and in cultured human pneumocytes demonstrated processing of SP-B by cysteine cathepsins and identified Ctsh as the very likely candidate [14], [19]. SP-B Western Blots of lung tissue lysates did not reveal significantly altered levels of SP-B precursor or SP-B processing intermediates in wild-type and Ctsh-deficient lungs. However, broncho-alveolar lavage (BAL) from *Ctsh* ^−/−^ mice contained lower levels of the fully processed 8.7 kDa SP-B (Fig. 4C). This result indicates reduced SP-B secretion by the alveolar epithelia and suggests an impaired functional quality of the lung surfactant of Ctsh knock-out mice. To further investigate this, we evaluated the composition and the surface activity of the surfactant which was isolated from the broncho-alveolar fluid (BALF) of *Ctsh^+/+^* and *Ctsh* ^−/−^ mice. We found no difference in the phospholipid concentration in the BALF (data not shown), however, phospholipid concentration was adjusted to 1 mg/ml in all further experiments. Using a pulsating bubble surfactometer the surface tension at the air–liquid interphase of bubbles that had not been pulsating was 36.4±1.4 mN/m at 10 s after the bubble's creation, a significantly higher value than in *Ctsh^+/+^* mice (Fig. 4D). The surface tension after 5 min of pulsation was not significantly different at maximal bubble radius, but at minimal radius surface tension was 11.0±1.7 mN/m and 18.7±0.9 mN/m for BALF from *Ctsh^+/+^* and *Ctsh* ^−/−^ mice, respectively (Fig. 4E,F). Together these results indicate that the reduced level of SP-B in the BALF of Ctsh-deficient mice impairs the tension reducing function of lung surfactant.

In summary, homozygous deletion of Ctsh by gene targeting does not result in a gross phenotype in the mice. However, in agreement with previous studies Ctsh is involved in SP-B processing and function of pulmonary surfactant derived from Ctsh null mice is impaired. This biochemical phenotype is not a complete phenocopy of inherited SP-B deficiency that results in severe respiratory failure at birth. It is tempting to speculate whether the slightly reduced frequency of Ctsh^−/−^ offspring from heterozygous mating may be caused by respiratory defects; however, we were not able to observe (and to analyze) dead newborns. It appears that the constitutive loss of Ctsh can be compensated functionally (but not by compensatory upregulation) by other cysteine or aspartic type proteases of type II pneumocytes, such as cathepsin C and cathepsin E, respectively. Using the newly developed Ctsh deficient mouse strain, a cooperation of cathepsins H and C in progranzyme B processing has been already established [16]. Hence, a network of proteases may maintain the level of SP-B processing and secretion that is needed to ensure sufficient surfactant and lung function in *Ctsh* ^−/−^ mice. However, these conclusions only hold true for the pathogen free conditions in which the mice were kept during this study. Cathepsin H knock-out mice may provide additional insights into the functions of this protease when challenged. In this regard, it has been shown recently that *Ctsh* ^−/−^ mice show less tumor burden and cancer invasiveness when analyzed in the context of the Rip1Tag2 model of pancreatic islet cancer [22]. Hence it will be worthwhile to investigate the respiratory tract of this knock-out mouse line when challenged by infections, sterile inflammations or cancerous growth.

## Materials and Methods

### Ethics statement

The generation and phenotype analysis of cathepsin H deficient mice in this study was performed in accordance to the German law for animal protection (Tierschutzgesetz) as published on May 25, 1998. According to this law the animal work was reviewed and approved by an ethics committee of the Regierungspräsdium (governmental regional board) Freiburg and given the project ID ‘G02/56 RP Freiburg’.

### Generation of mice with targeted disruption of cathepsin H (Ctsh)

Part of exon 5 through intron 9 of the Ctsh gene were replaced by a targeting vector comprising IRES, lacZ-Reporter and a G418 resistance cassette (neo^r^) using homologous recombination in HM1-mouse embryonic stem cells (Fig. 1). G418-resistant HM1- cell clones (129P2/OlaHsd background) were screened by Southern blot analysis of genomic DNA, which was digested with SacI and hybridized with the external probe (Fig. 1B). Mutated ES cells were microinjected into blastocysts of C57BL/6N females. The resulting chimeras were used to generate heterozygous mutant offspring on a mixed C57BL/6N and the 129P2/OlaHsd genetic background. Mice were backcrossed onto the C57BL/6N for 10 generations. Mice were bred and maintained under specific-pathogen-free conditions.

### Genotyping

Routine genotyping of Ctsh deficient mice is performed on genomic DNA by PCR. The primer pair Ctsh-fw / Ctsh-rev amplifies 426 base pairs of the wild-type allele that is deleted in the mutant: Ctsh-fw: 5′ TAA-ATG-GGC-TAG-TGA-ATG-CTG-ACG 3′; Ctsh-rev: 5′ TGA-ATC-TGG-AGT-TTG-GAG-GGT-AGT 3′. The primer neo^r^ anneals in the 3̀end of the G418 resistance cassette while primer Ctsh intron 9-rev is located in the not deleted part of intron 9. Thus this primer pair will amplify the mutated alleles as an 205 bp fragment : neo^r^ 5′ ATC-GCC-TTC-TAT-CGC-CTT-CTT 3̀; Ctsh intron 9-rev: 5′ CAC-CCC-ATG-ATT-CCT-TCC-TG 3̀. PCR annealing temperature of 56°C for 30 seconds and elongation at 72°C for one minute are recommended for a total of up to 35 cycles.

### qRT PCR

Total RNA was isolated from frozen lung tissue samples with RNAeasy Mini kit (Qiagen) and reverse transcribed using iSript cDNA Synthesis kit (Bio-Rad). Transcripts were quantified using Platinum SyBR® Green qPCR SuperMix (Invitrogen) and the following primer pairs: Ctsc, 5′ ACC TGG GTG TTC CAG GTG GGC CC3′ (fw.) and 5′GCC CGG AAT TGC CCA GCT CGT CG3′ (rev.); Ctse, 5′CAG TCC GAC ACA TAC ACG3′ and 5′TGC CCT GGC TCC TTG AC3′ (rev.); SP-A1, 5′TGC AGG CTC TGT GTG CGG GGA TCT3′ (fw.) and 5′CAG GGA TCC CAG GGC TTC CGG CA (rev); SP-B, 5′AGC AAC AGC TCC CCA TTC CCC TGC C3′ (fw.) and 5′CCA CCA CCA GGG GTA CCA CGT GGC3′ (rev.); SP-C, 5′TGA CTA CCA GCG GCT CCT GAC GGC3′ (fw.) and 5′GTG GGT GTG GAG GGC TTG GCC TGG3′ (rev.).

### Northern blot

For detection of Ctsh mRNA by Northern blots, total RNA of kidney and liver from adult mice was prepared according to a standard protocol [23]. Subsequently 5 µg total RNA was separated in a formaldehyde agarose gel and processed as described previously [24]. Filters were hybridized with [α-32P]dCTP labelled probes of Ctsh exon 3 and a cDNA fragment of mouse β-actin.

### Enzymatic activity

Ctsh aminopeptidase activity was measured by degradation of synthetic substrate H-Arg-4-Methyl-Coumarin-7-Amid (AMC). A crude organelle fraction was obtained by differential centrifugation of Dounce-homogenized tissues and organelles were broken by ultra-sound in 50 mM of phosphate buffer (pH 6.0) containing 2.5 mM EDTA/ 2.5 mM DTT. H-Arg-AMC substrate (50 µM) was added and release of fluorescent AMC (excitation 359 nm/emission 540 nm) was monitored for 30 min at 37°C.

### Western blot

Aliquots of the BALF (3 µg protein) or crude organelle fractions (10 µg protein) were separated using 4-12% NuPage® Bis-Tris Gel (Novex / Invitrogen, Carlsbad, USA) and MES buffer and blotted to nitrocellulose membranes (Hybond ECL, Amersham Pharmacia Biotech, Little Chalfont, U.K.). The membranes were blocked using non-fat dry milk (16 h, RT) and incubated with affinity purified SP-B antiserum (kindly donated by Dr. J.A. Whitsett (Cincinatti, OH)) or with.affinity purified cathepsin H antiserum (kindly donated by Dr. E. Weber (Halle, Germany). The immunoreaction was detected using polyclonal antisera directed against rabbit or mouse IgG conjugated to horse radish peroxidase and ECT (Novex / Invitrogen, Carlsbad, USA) as substrate.

In order to detect proSP-B frozen lung tissue samples were lysed with TritonX lysis buffer (0.2% TritonX-100 in PBS). To detect Ctsc and Ctse sodium acetate lysis buffer (100mM sodium acetate, 1mM EDTA, 0.05% Brij) was used. Proteins (10 µg) were separated on 15% Bis-Tris gels and blotted to PVDF membranes (Amersham). Membranes were blocked with 3% powdered milk (for Ctsc, and Ctse) or 4% BSA (for SP-B). For immunodetection membranes were probed with a 1∶500 dilution of anti-mouse Ctsc or anti-mouse Ctse antibody (R&D Systems AF1034, AF1130), or anti-SP-B antibody (Millipore ABS21) and HRP conjugated anti-goat (Sigma A5420) or anti-rabbit (Biorad 172-1019) secondary antibodies.

### Histology, Immunohistochemistry and β-galactosidase (LacZ) staining

The Lac-Z reporter in the gene targeting construct provides direct visualization target gene expression in tissues of gene targeted mice. Fixation of the lungs was by lacZ-fixation solution (0.4 ml 25% glutaraldehyde; 1.25ml 0.2M EGTA (pH 7.3); 5.0ml 1M MgCl2; 43.0 ml PBS) for 2h at 4°C. After 3x5 min PBS washes and the samples were incubation in 20% sucrose in PBS for 16 h at 4°C. The dehydrated tissues were embedded in OCT (water soluble glycol's and resins compound) by freezing in liquid nitrogen. Cryosections (10 µm tickness) were post-fixed in 0.2% glutaraldehyde / PBS for 10 min at room temperature followed 3x5min washes in lacZ-buffer composed of 0.5ml 1M MgCl_2_; 0.25 ml 10% sodium-deoxycholate; 2.5ml 2% Nonidet-P40; 265 ml PBS. Detection of lacZ (i.e. β-galactosidase activity) section was achieved by an overnight incubation in 87.5ml lacZ buffer supplemented with 2.5 ml X-gal (i.e. bromo-chloro-indolyl-galacto-pyranoside dissolved at 40mg/ml in DMSO) and 5 ml 0.1M potassium-ferricyanide at 37°C in the dark. The stained sections were observed und photographed under digital optical microscope.

For immunodetecton of Ctsh, paraffin embedded lung tissue sections were incubated with a 1∶40 dilution of anti- mouse Ctsh antibody (R&D Systems AF1013) followed by detection with the Vectastain Elite ABC kit (Vector Laboratories) and 3,3-DAB and nuclear staining with hematoxylin.

### Broncho-alveolar lavage (BAL)

BAL was performed by cannulating the trachea and infusing the lungs with 2×0.8 ml cold sterile PBS/2 mM EDTA. The broncho-alveolar lavage fluid (BALF) was retrieved by gentle aspiration. The BALF was pooled and the cells were transferred to a microscope slide by cytospin (10 min, 400 xg). The supernatants were collected and frozen at –80°C.

### Surfactant aggregate separation and evaluation with pulsating bubble surfactometer

The cell-free supernatant was centrifuged at 48,000 xg for 60 min at 4°C to pellet large surfactant aggregates (LA). The supernatant, containing small surfactant aggregates (SA), was removed and the LA pellet was resuspended in Ringer's solution. The phospholipid contents of the LA pellets and the SA supernatants were determined as described [25]. By adding Ringer's solution, the phospholipid concentration of the LA suspension was adjusted to 1 mg/ml. Surface activity of BALF was measured with a pulsating bubble surfactometer (Electronetics, Buffalo, NY) [26]. For the pulsating bubble surfactometer, 40 µl of the LA suspension, which had been given a phospholipids concentration of 1 mg/ml, were used for filling the sample chamber with a micropipet. The surface tension used for statistical analysis of this study was the value at minimal bubble size (γ_min_) registered after 5 min of pulsation at a rate of 20 cycles/min and at a temperature of 37°C. Before starting, bubble pulsation adsorption rate was evaluated by determining surface tension 10 s after formation of a bubble (γ_ads_).
